# Evaluation of conservative treatment and timing of surgical intervention for mild forms of cervical spondylotic myelopathy

**DOI:** 10.3892/etm.2013.1224

**Published:** 2013-07-16

**Authors:** LING-DE KONG, LING-CHEN MENG, LIN-FENG WANG, YONG SHEN, PAN WANG, ZI-KUN SHANG

**Affiliations:** Department of Spine Surgery, The Third Hospital of Hebei Medical University, Shijiazhuang, Hebei 050051, P.R. China

**Keywords:** cervical myelopathy, conservative treatment, surgical treatment, prognosis

## Abstract

The optimal management approach for patients with mild forms of cervical spondylotic myelopathy (MCSM) has not been well established. The aim of the present study was to investigate the outcome of conservative treatment, identify prognostic factors and provide evidence for the timing of surgical intervention. A total of 90 patients with MCSM attending hospital between February 2007 and January 2009 were prospectively enrolled. Initially, all patients received conservative treatment and were followed up periodically. When a deterioration in myelopathy was clearly identified, surgical treatment was conducted. Clinical and radiological factors correlating with the deterioration were examined, and final clinical outcomes were evaluated using the Japanese Orthopedic Association (JOA) score. At the end of January 2012, follow-ups of >3 years were completed. Seventy-eight patients were available for data analysis. Only 21 patients (26.9%) deteriorated and underwent surgery thereafter (group A), while the remaining 57 patients (73.1%) were treated conservatively throughout (group B). Statistical analysis revealed that segmental instability and cervical spinal stenosis were adverse factors for the prognosis of conservative treatment. Although the JOA scores of the patients in group A declined initially, following surgical intervention, no significant differences were identified in JOA scores between the two groups at the time of the final follow-up (P=0.46). In summary, conservative treatment is effective in MCSM patients. Patients with segmental instability and cervical spinal stenosis have a tendency to deteriorate, but conservative treatment remains the recommendation for the first action. If the myelopathy deteriorates during conservative treatment, timely surgical intervention is effective.

## Introduction

Cervical spondylotic myelopathy (CSM) is a degenerative disease of the cervical spine in which the severity of clinical signs and symptoms depends on the pathophysiology of the spinal cord. Since osteoarthritic degeneration of the cervical spine may provoke irreversible damage to the spinal cord and delays in surgical treatment tend to result in a poor outcome (1–3), early decompression surgery is usually recommended for patients with a definite diagnosis of moderate or severe CSM (4–6). However, in patients with mild, non-progressive or slowly progressive CSM, a relatively benign natural course has been observed (7,8) and the superiority of surgical treatment over conservative treatment has not been established (9–11). It appears that conservative treatment is the preferred choice for these patients since it avoids the complications of surgery and is less expensive (12,13). However, even with the same treatment protocols, the outcome of conservative treatment for mild forms of CSM (MCSM) varies between individuals. Although efforts have been made to determine prognostic factors for the deterioration of MCSM, results remain highly controversial (7,8,14–16). Furthermore, for MCSM patients with adverse prognostic factors, little is known with regard to the optimal timing of surgery. Surgeons are faced with a dilemma as to whether surgery should be performed as early as possible or only after conservative treatment has failed.

With the aim of providing evidence-based guidelines for the effective treatment of MCSM, the present prospective study was conducted to investigate the clinical outcome of conservative treatment, identify factors associated with the prognosis of conservative treatment and to verify whether it is too late to perform surgical procedures after conservative treatment fails.

## Materials and methods

### Inclusion and exclusion criteria

Following approval from the ethics committee of The Third Hospital of Hebei Medical University (Shijiazhuang, China), 90 MCSM patients attending The Third Hospital of Hebei Medical University (Shijiazhuang, China) between February 2007 and January 2009 were prospectively enrolled in this study, and informed consent was obtained from all patients. Inclusion criteria for patients were as follows: objective clinical findings of CSM, for example, increased deep tendon reflexes, positive pathological reflexes and abnormal sensory disturbances; correlative spinal cord compression on magnetic resonance imaging (MRI); a Japanese Orthopedic Association (JOA) score (Table I) of ≥13; a recent non-progressive process; and consent provided for the recommended treatment plan. Patients with ossification of the posterior longitudinal ligament, traumatic cervical myelopathy, motor neurone disease, multiple sclerosis, progressive polyarthritis, congenital anomalies and vitamin B_12_ deficiency were excluded from the study.

### Treatment protocol

Each patient was hospitalized and received treatment according to the following protocol. The initial treatment comprised continuous cervical traction [Good-Samaritan traction (17)] in which the neck of the patient was placed in a position of slight flexion for 8 h per day for ~2 weeks. Regardless of the outcome of cervical traction, patients were discharged. Hyper-extended or hyper-flexed neck positions, slips and falls, intense exercise and other potentially dangerous activities in daily life were avoided at home. Patients were followed up periodically every three months and advised to visit the hospital immediately if their symptoms became exacerbated. Surgery was performed on patients who experienced a deterioration of myelopathy that resulted in JOA scores of <13 with a reduction of ≥2 points.

### Surgical protocol

The procedures included anterior and posterior approaches, the choice of which depended on the cervical alignment and the levels and sources of compression. Anterior discectomy followed by autologous bone grafting and cervical plate fixation were adopted for patients with 1- or 2-level compression, particular those with greater compression on the anterior side. In patients exhibiting preserved cervical lordosis and >3-level canal stenosis, C3 to C6 (or C7) laminoplasty was performed. In the case of significant compression on the posterior side, laminoplasty was also selected, even if the patients exhibited <3-level compression. All surgeries were performed within one month of deterioration of myelopathy. At the end of the study, all surgically treated patients were followed up for ≥1 year postoperatively.

### Outcome assessments

**Figure 1:**
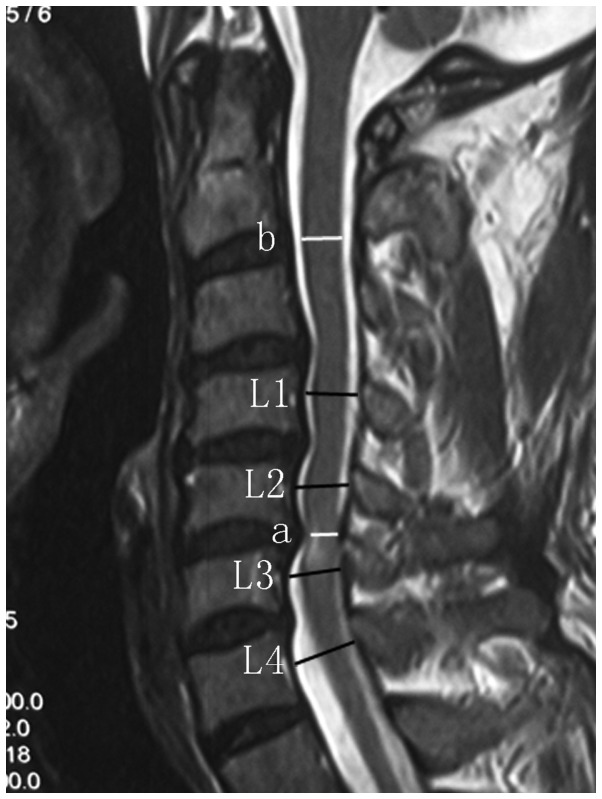
Measurement of diameters of the cerebrospinal fluid (CSF) column and spinal cord compression on T2 sagittal MRI. L1–L4 were diameters of the CSF at the mid-vertebra level from C4 to C7. Diameter of the CSF column (mm) = (L1+L2+L3+L4)/4. a and b are the spinal cord diameters of narrowest part and the C2/3 intervertebral level. Spinal cord compression (%) = a/b.

In order to analyze the prognostic factors, relevant clinical factors, including gender, age and duration of disease, were collected from the records of the patients. On the first visit to the hospital, all patients underwent plain antero-posterior, lateral and extension-flexion radiographs with a tube-to-film distance of 120 cm. All patients underwent high-resolution MRI with a 1.5-T imager (Siemens Magnetom Symphony; Siemens, Berlin, Germany). The conditions for the MRI study were as previously described (3). Plain radiographs and MRI yielded imaging parameters as follows: The C2–C7 angle was measured using the Cobb method, which was determined by the inferior margin of C2 body and the inferior margin of C7 body on lateral radiographs; segmental instability was considered to be present if >2 mm of slippage displacement was observed on extension-flexion radiographs at the compression-affected levels (18). The sagittal diameters of the cerebrospinal fluid (CSF) column were measured at the mid-vertebra level on T2 sagittal MRI from C4 to C7, and the mean value was calculated (Fig. 1). The extent of spinal cord compression was defined by the ratio of the spinal cord diameter of the narrowest part to that of the C2/3 intervertebral level using sagittal images on T2-weighted MRI (Fig. 1). The number of compression-affected levels on T2-weighted MRI was also recorded. Data measurements were performed twice by a single observer and the mean value was used for analysis.

To investigate treatment outcomes, JOA scores at the time of the initial visit and final follow-up were analyzed. For patients who neurologically deteriorated and therefore underwent surgery, JOA scores at the time of conversion to surgery were also evaluated.

To determine which prognostic factors correlated with failed conservative treatment, each of the clinical and radiological factors was compared between patients who were treated conservatively throughout and those who had undergone surgery by the final follow-up.

### Statistical analysis

A parametric analysis was performed using the Mann-Whitney U test. Categorical variables were analyzed using the Fisher's exact test. The Statistical Package for the Social Sciences (version 13.0 for Windows; SPSS, Inc., Chicago, IL, USA) was used for the statistical analysis. P<0.05 was considered to indicate a statistically significant difference.

## Results

**Figure 2:**
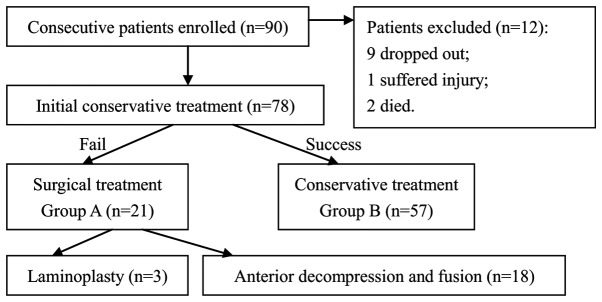
Treatment profile in of patients with mild forms of cervical spondylotic myelopathy (MCSM) in the present study.

A total of 90 patients with MCSM were enrolled in this study. By the end of January 2012, nine patients had withdrawn from the study, one patient was affected by an acute spinal cord injury and two patients had succumbed to unrelated causes (Fig. 2). At the final follow-up, 78 (45 male and 33 female) of 90 patients (follow-up rate, 86.7%) were investigated. The average age at first visit was 57.8 years (range, 37–71 years). The average follow-up period was 40 months (range, 36–56 months). The mean JOA scores at the time of the first and the last follow-up were 14.1 points (range, 13–16 points) and 14.0 points (range, 10–16 points), respectively (Table II).

The 78 patients were divided into two groups according to the results of conservative treatment. The 21 patients who had gradual reductions in JOA scores to a mean of 2.9 points (range, 2–5 points) and underwent surgery were group A, while the remaining 57 patients who were treated conservatively throughout were group B. Among the 21 patients in group A, 18 patients underwent anterior decompression and fusion and three underwent laminoplasty. There were no incidences of infections, cervical fluid leakage, esophageal or tracheal ruptures and neurological deterioration. Transient recurrent laryngeal nerve palsy was observed in one patient without further management. Three patients reported axial pain, which subsided gradually in 1–2 months without any treatment.

The prognostic factors correlating with the outcome of conservative treatment are displayed in Table III. The mean diameter of the CSF column in group A was significantly smaller than that in group B (10.7±1.8 vs. 12.1±1.2 mm, P=0.02). Additionally, nine of 21 (42.9%) patients exhibited segmental instability in group A compared with only eight of 57 (14.0%) patients in group B. This difference was statistically significant (P=0.01). No significant differences were identified between the two groups with regard to age, gender, duration of disease, C2–C7 angle, signal intensity changes on MRI, the number of compression-affected levels and the degree of spinal cord compression.

**Figure 3:**
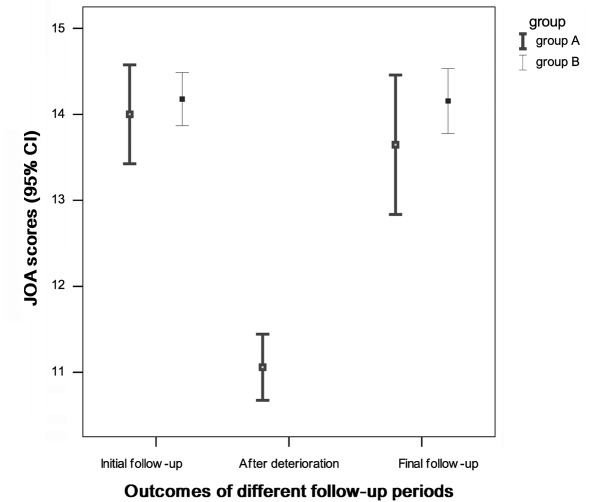
Changes in Japanese Orthopedic Association (JOA) scores.

Fig. 3 shows the mean JOA scores at different follow-up periods. On the first visit to the hospital, the mean JOA scores were 14.0±1.1 in group A and 14.2±1.0 in group B, and no significant difference was identified between the two groups (P=0.62). Although the mean JOA score of group A decreased to 11.1±0.8 as the condition deteriorated, following timely surgical intervention, the final mean JOA scores of the two groups (13.4±2.5 in group A and 14.2±1.3 in group B, P=0.46) were not observed to be significantly different.

## Discussion

The present study revealed a satisfactory outcome of conservative treatment in MCSM patients; only 21 of 78 (26.9%) patients experienced a deterioration of myelopathy during a >3-year follow-up. This demonstrated that in the majority of the MCSM patients, neurological function either improved or was non-progressive. This finding is consistent with a number of previous reports. Oshima *et al*(7) conducted a retrospective study with a mean follow-up period of 78 months to investigate the natural course of MCSM; 16 of 43 (37.2%) patients exhibited a deterioration in motor function and underwent decompression surgery. Following Kaplan-Meier survival analysis, it was revealed that 82 or 56% of patients in the study did not require surgery five or 10 years after initial treatment, respectively. Sumi *et al*(8) performed a prospective cohort study that only focused on MCSM and had a 5–12-year follow-up period, in which deterioration in myelopathy was observed in 14 of 55 (25.5%) patients, whereas 41 of 55 (74.5%) patients maintained mild myelopathy without deterioration through the follow-up period.

The selection of patients who will truly benefit from conservative treatment is challenging to surgeons. It is therefore critical to investigate which prognostic factors aggravate MCSM. A number of predicting factors that may affect the surgical outcomes of CSM have been reported, including age (1,19), duration of disease (1), cervical curvature (20), signal intensity changes in MRI (3,21) and extent of spinal cord compression (22). Despite this, studies reporting the prognostic factors of conservative treatment for MCSM are limited, and the conclusions controversial. Shimomura *et al*(14) reported that circumferential spinal cord compression in the maximum compression segment in axial MRI was the only prognostic factor for MCSM. Subsequently, Sumi *et al*(8) further followed the same patients for >5 years and reported that the presence of angular-edged deformities of the spinal cord in T1-weighted axial MRI is another risk factor that aggravates myelopathy. Yoshimatsu *et al*(15) analyzed the results of conservative treatment for CSM in 69 cases and concluded that the disease duration correlated significantly with the clinical outcome. However, Oshima *et al*(7) indicated that a large range of motion (ROM) and segmental instability at the narrowest canal are considered to be adverse prognostic factors.

Among several parameters considered in the present study, the diameter of the CSF column and the existence of segmental instability were identified to be statistically significant. Narrowing of the spinal canal has been demonstrated to be a major risk factor for CSM (23,24). We hypothesized that the presence of cervical spinal stenosis may expose individuals to a greater risk of deterioration of MCSM. Historically, the evaluation of cervical spinal stenosis has been based on the Pavlov ratio in plain lateral radiographs (25). However, the Pavlov ratio may not reflect the impact of soft tissue on MCSM, including hypertrophy of the ligamentum flavum. We consider the diameter of the CSF column on MRI to more accurately reflect the space in the cervical canal. Furthermore, the presence of segmental instability is another risk factor, which indicates that the exacerbation of MCSM is affected not only by static compression, but also by dynamic factors that inflict repeated minor traumas on the spinal cord.

Although efforts have been made to identify prognostic factors, it is difficult to predict whether a given factor will be responsible for deterioration in a particular instance. Consequently, it remains to be determined whether patients with MCSM and adverse prognostic factors should be surgically treated as soon as possible to avoid potential deterioration, even if their symptoms and signs are moderate. Furthermore, if non-surgical therapy is adopted, there may be a risk of prolonged conservative treatment resulting in a lost opportunity to obtain satisfactory outcomes after surgery. In an attempt to clarify these points, patients in group A were further followed up postoperatively for ≥1 year. A poor outcome would indicate that patients with adverse prognostic factors require referral to surgery prior to deterioration. By contrast, the absence of a poor outcome would indicate that surgery may be postponed. The results demonstrated that surgical outcomes were relatively good, suggesting it was not too late to perform surgery following the failure of conservative treatments. Thus, although they have a tendency to undergo deterioration, MCSM patients presenting with segmental instability or cervical spinal stenosis may initially be treated conservatively with a close follow-up. Surgical procedures may subsequently be selected when deterioration of myelopathy is clearly identified. However, surgery should be performed in a timely manner since CSM may progress rapidly and a long period of moderate or severe CSM may result in poor prognosis (1–3).

In summary, in this study, it was observed that MCSM patients treated conservatively showed a relatively benign clinical course. Segmental instability and cervical spinal stenosis were factors correlating with a poor prognosis. For patients with adverse prognostic factors, conservative treatment remains the recommendation for the first choice action. If conservative treatment fails, timely surgical intervention is likely to be successful.

However, this study had several limitations. Firstly, the JOA score system was the only criteria used to select MCSM patients and assess clinical outcomes. The scores were acquired based on the perspective of an investigator and thus were vulnerable to subjectivity. Secondly, patients were followed up for only three years; this relatively short follow-up time weakens the ability of the study to assess the clinical course of MCSM. Consequently, future studies involving reliable, validated assessment tools and long-term follow-up periods are required to further confirm the present findings.

## Figures and Tables

**Table I:** Japanese Orthopaedic Association (JOA) scores for assessment of cervical myelopathy.

Category	Score (points)
Motor function of the upper extremity	
Unable to eat with either chopsticks or a spoon	0
Able to eat with a spoon, but not with chopsticks	1
Able to eat with chopsticks, but inadequately	2
Able to eat with chopsticks, but awkwardly	3
Normal	4
Motor function of the lower extremity	
Unable to walk	0
Needs a cane or other walking aid on flat ground	1
Needs walking aid only on stairs	2
Able to walk unaided, but slowly	3
Normal	4
Sensory function	
Upper extremity	
Apparent sensory disturbance	0
Minimal sensory disturbance	1
Normal	2
Lower extremity	
Apparent sensory disturbance	0
Minimal sensory disturbance	1
Normal	2
Trunk	
Apparent sensory disturbance	0
Minimal sensory disturbance	1
Normal	2
Bladder function	
Urinary retention or incontinence	0
Severe dysuria (sense of retention)	1
Slight dysuria (pollakiuria, retardation)	2
Normal	3

The score in a normal subject is the total of the highest scores: (I + II + III + IV) = 17 points.

**Table II:** Baseline demographic characteristics.

Variable	Value
No. of patients	78
No. of deteriorated patients	21
Gender (male:female)	45:33
Age (years)	37–71 (57.8)
Follow-up period (months)	36–56 (40.0)
JOA scores of initial follow-up	13–16 (14.1)
JOA scores of final follow-up	10–16 (14.0)

Values in parentheses are mean values. JOA, Japanese Orthopedic Association.

**Table III:** Risk factors of neurological deterioration.

Factor	Group A (n=21)	Group B (n=57)	P-value
Age (years)	58.8±11.3	57.5±10.5	0.62
Gender (male:female)	13:8	32:25	0.80
Duration of disease (months)	23.3±19.2	19.3±15.0	0.70
C2–C7 angle (degrees)	9.9±10.7	11.6±10.7	0.61
Segmental instability (yes:no)	9:12	8:49	0.01
Spinal cord intensity changes (yes:no)	14:7	26:31	0.13
Compression-affected levels (no.)	1.4	1.3	0.53
Diameter of the CSF column (mm)	10.7±1.8	12.1±1.2	0.02
Spinal cord compression (%)	78.2±6.3	75.3±8.1	0.19

Measurement results are expressed as the mean ± SD. Group A, deterioration after conservative treatment; Group B, no deterioration after conservative treatment; CSF, cerebrospinal fluid.
